# The AtCRK5 Protein Kinase Is Required to Maintain the ROS NO Balance Affecting the PIN2-Mediated Root Gravitropic Response in Arabidopsis

**DOI:** 10.3390/ijms22115979

**Published:** 2021-06-01

**Authors:** Ágnes Cséplő, Laura Zsigmond, Norbert Andrási, Abu Imran Baba, Nitin M. Labhane, Andrea Pető, Zsuzsanna Kolbert, Hajnalka E. Kovács, Gábor Steinbach, László Szabados, Attila Fehér, Gábor Rigó

**Affiliations:** 1 Biological Research Centre (BRC), Institute of Plant Biology, Eötvös Loránd Research Network (ELKH), H-6726 Szeged, Hungary; cseplo.agnes@brc.hu (Á.C.); zsigmond.laura@brc.hu (L.Z.); andrasinorbi@gmail.com (N.A.); abu.baba@slu.se (A.I.B.); kovacs.hajnalka.eva@gmail.com (H.E.K.); steinbach.gabor@brc.hu (G.S.); szabados.laszlo@brc.hu (L.S.); feher.attila@brc.hu (A.F.); 2 Umeå Plant Science Centre, Department of Forest Genetics and Plant Physiology, Swedish University of Agricultural Sciences, SE-901 83 Umeå, Sweden; 3 Department of Botany, Bhavan’s College Andheri West, Mumbai 400058, India; nitin.labhane@bhavans.ac.in; 4 Department of Plant Biology, University of Szeged, 52. Középfasor, H-6726 Szeged, Hungary; petoandrea@gmail.com (A.P.); ordogne.kolbert.zsuzsanna@szte.hu (Z.K.); 5 Food Chain Safety Center Nonprofit Ltd., H-1024 Budapest, Hungary; 6 Budapest, Kossuth Lajos Sugárút, 72/D, H-6724 Szeged, Hungary; 7 Cellular Imaging Laboratory, Biological Research Centre, Eötvös Loránd Research Network, H-6726 Szeged, Hungary

**Keywords:** auxin transport, Calcium-Dependent Protein Kinase-Related Kinase (CRK), reactive oxygen species, superoxide anion, hydrogen peroxide, nitric oxide, paraquat, oxidative stress, root gravitropism, Arabidopsis

## Abstract

The Arabidopsis AtCRK5 protein kinase is involved in the establishment of the proper auxin gradient in many developmental processes. Among others, the At*crk5-1* mutant was reported to exhibit a delayed gravitropic response via compromised PIN2-mediated auxin transport at the root tip. Here, we report that this phenotype correlates with lower superoxide anion (O_2_^•−^) and hydrogen peroxide (H_2_O_2_) levels but a higher nitric oxide (NO) content in the mutant root tips in comparison to the wild type (AtCol-0). The oxidative stress inducer paraquat (PQ) triggering formation of O_2_^•−^ (and consequently, H_2_O_2_) was able to rescue the gravitropic response of At*crk5-1* roots. The direct application of H_2_O_2_ had the same effect. Under gravistimulation, correct auxin distribution was restored (at least partially) by PQ or H_2_O_2_ treatment in the mutant root tips. In agreement, the redistribution of the PIN2 auxin efflux carrier was similar in the gravistimulated PQ-treated mutant and untreated wild type roots. It was also found that PQ-treatment decreased the endogenous NO level at the root tip to normal levels. Furthermore, the mutant phenotype could be reverted by direct manipulation of the endogenous NO level using an NO scavenger (cPTIO). The potential involvement of AtCRK5 protein kinase in the control of auxin-ROS-NO-PIN2-auxin regulatory loop is discussed.

## 1. Introduction

Environmental signals (light, water, mechanical touch, gravitropic stimuli, etc.) affect plant development in various ways, including the determination of the direction of organ growth. The differential growth responses reorienting plant organs in response to directional environmental cues are defined as tropic plant movements (plant tropisms). As it was laid down in the Cholodny–Went hypothesis almost a century ago, the regulated transport of the plant hormone auxin controlling the differential elongation of cell files proximal and distal to the signal source has a central role in tropic responses [1]. Although it has been questioned that the theory is as universal and simple as was originally thought, molecular biology experiments have confirmed the significance of auxin redistribution in many tropic responses [2,3], including the gravitropic response of roots [4].

Alterations in the direction of the gravity vector of the roots are sensed by specific cells of the root columella called statocysts [5]. The initial steps of the signalling pathway leading to altered auxin transport and signalling are not yet fully understood but might include changes in the concentrations of inositol 1,4,5-triphosphate (IP3) and cytoplasmic Ca^2+^ [5]. At the end, the gravitropic curvature of the root is the consequence of the asymmetrically distributed auxin, causing differential cell elongation [1,4,5].

In the dicot Arabidopsis, the cell-to-cell transport of auxin is mediated by AUX1/LIKE-AUX1 (AUX/LAX) auxin influx transporters [6], PIN-FORMED (PIN) auxin efflux transporters [7], and PGP-glycoprotein/ATP binding cassette protein subfamily B transporters (ABCB transporter family members) [8]. While the H^+^/auxin symporters of the AUX1/LAX family facilitate the uptake of indole-acetic acid anions (IAA^−^) from the apoplast into the cytoplasm, the plasma-membrane-associated members of the PIN family are responsible for the directional efflux of IAA^−^ from the cell and are supported by ABCB transporters [9]. The main sites of auxin production are at the shoot tip. The shoot-derived auxin is transported towards the root tip where it accumulates at the quiescent centre and in the upper tiers of the root-cap columella and is then redistributed radially to peripheral cell files, transporting it basipetally towards the root elongation zone. In the various regions of the root tip, the PIN proteins display well-defined polar localization in the cell’s plasma membrane (PM) in agreement with the direction of the auxin flow [10]. For example, PIN2 is localized in the PM membranes of the epidermal and cortical cells as well as in the lateral root cap (LRC) cells [10,11,12]. The polar localization of PIN family members in the PM is dynamic due to the continuous endocytic recycling of these proteins [13,14]. The vesicular trafficking and turnover of the PIN proteins is controlled, among others, by the auxin itself enforcing its own directional transport [13].

AtPIN2 has an important role in the gravitropic bending of Arabidopsis roots [11,12]. It is the PIN2-dependent asymmetrical auxin distribution in the lower and the upper tiers of epidermal cells in the horizontal root that evokes differential cell elongation and the bending of the root tip towards gravitropic stimuli [11,15,16]. PIN2 turnover is differentially regulated at the two sides of the gravistimulated root, resulting in its accumulation at the lower epidermis/cortex where it augments the auxin level that inhibits cell elongation [11,15,16]. The asymmetry in PIN2 abundance is reinforced by auxin controlling PIN2 transcription, internalization, vacuolar targeting, and degradation [11,15]. The membrane-targeting and stability of PIN proteins are also controlled by their phosphorylation status [17,18]. The gravitropic response of Arabidopsis roots were shown to be reciprocally regulated phosphorylation and dephosphorylation of PIN2 by the PINOID (PID) protein kinase and the ROOTS CURL IN NAPHTHYLPHTHALAMIC ACID1 (RCN1) protein phosphatase, respectively, [19].

Signals like Ca^2+^ [20,21,22,23], phosphatidylinositol-4,5-bisphosphate (PtdIns(4,5)P(2) [24]), inositol 1,4,5-trisphosphate (InsP_3_) [25,26,27], apoplastic pH [21,28], nitric oxide (NO) [29,30], and reactive oxygen species (ROS) [31,32,33] were also shown to be important elements of the auxin-dependent root gravitropism.

According to Perera [26,27], gravitropism stimulates the transient generation of InsP_3_, which then supports the opening of an IP_3_-induced Ca^2+^ channels to increase intracellular Ca^2+^ concentrations. The Ca^2+^ signal can be transduced towards the gravitropic response, among others, by Ca^2+^/CALMODULIN-DEPENDENT PROTEIN KINASE-RELATED KINASES (CRKs) [34,35]. CRKs are Ser/Thr protein kinases with diverse functions in development and stress adaptation [36]. The Arabidopsis CRK subfamily consists of eight members [36]. T-DNA insertion in any of the AtCRK genes was shown to delay the gravitropic responses of roots as well as hypocotyls [34,37]. The role of the AtCRK5 protein kinase was studied in more detail in our laboratory [34,35,37,38]. The AtCRK5 protein kinase is active in most *Arabidopsis* organs [35]. It is associated with the plasma membrane due to its N-terminal myristoylation site [35]. The kinase was found to be involved in the establishment of proper auxin gradient during several developmental processes such as embryo development [38], hypocotyl hook establishment [37], and the root gravitropic response [35]. AtCRK5 is capable of phosphorylating the PIN auxin efflux carriers, including PIN2, PIN3, PIN4, and PIN7, that might affect their turnover and, in consequence, the auxin distribution [35,37,38]. In the transition zone of At*crk5-1* mutant roots, PIN2 was found to be depleted from the apical membranes of epidermal cells and was delocalized form the basal to the apical membrane of cortex cells [35]. This resulted in facilitated auxin transport from the meristem towards the elongation zone inhibiting root growth and the gravitropic response [35]. Here, we describe that the AtCRK5 protein kinase is also required to maintain the redox homeostasis at the root tip that contributes to the proper graviresponse of Arabidopsis roots.

The various types of ROS (including O_2_^•−^ and H_2_O_2_) are distributed along different gradients in the roots where they affect cell division and elongation [39]. Root gravistimulation was shown to lead to the transient and asymmetric generation of ROS at the convex endodermis of maize roots [31]. The auxin-induced production of ROS was found to be dependent on phosphatidylinositol 3-kinase activity and thus inositol trisphosphate (InsP_3_) accumulation [25]. The unilateral application of H_2_O_2_ resulted in the bending of roots, even if auxin transport was inhibited and unilateral exogenous auxin application triggered transient ROS accumulation, indicating that ROS act downstream of auxin in the gravitropic response [31]. However, the relationship between ROS and auxin in the roots is rather complex. Exogenous auxin reduced the O_2_^•−^ production and inhibited the growth of maize roots [40]. ROS were hypothesized to attenuate IAA signalling to allow for the reset of auxin sensitivity under normal conditions [41], while stress-triggered high ROS levels were reported to downregulate auxin transport by decreasing the abundance of PIN auxin efflux carriers at the PM [42]. Specifically, PIN2 endocytic recycling has been found to be inhibited by elevated H_2_O_2_ levels [42].

Besides ROS, NO is also in crosstalk with auxin during root growth control [43,44,45,46]. NO might act downstream or upstream of auxin signalling in the various root developmental processes, including primary root growth, adventitious root organogenesis, lateral root emergence, and root hair formation [44]. Exogenous NO treatment reduced PIN1-mediated acropetal auxin transport that resulted in root meristem defects in Arabidopsis [47]. NO was shown to attenuate auxin signalling due to S-nitrosation of the auxin receptor TRANSPORT INHIBITOR RESPONSE 1 (TIR1) [48]. In NO-deficient mutants or NO-depleted roots, the biosynthesis, transport, and signalling of auxin are disturbed hindering proper stem cell niche organisation and meristem function [46]. Of note: the NO depleted roots exhibit elevated levels of reactive oxygen species (ROS) [46]. The crosstalk between NO and ROS is well demonstrated during various developmental processes including the organization of the root system [49]. Altogether, the experimental observations indicate that keeping the NO level in an optimal range is required for sustained root growth and development [50]. NO was implicated as a downstream element of auxin signalling during the gravitropic bending in soybean roots [29]. It was also demonstrated that the transient and asymmetric accumulation of endogenous NO contributes to the early gravitropic response in Arabidopsis roots [30]. NO was shown to promote the PM relocalization of PIN2 as part of the gravitropic response in the root epidermal cells [30].

Here, we present experimental data supporting the view that the AtCRK5 protein kinase is involved in the auxin–ROS–NO crosstalk. Its potential role in the feedback regulation of PIN2-dependent auxin transport during the gravitropic growth of Arabidopsis roots is discussed.

## 2. Results

### 2.1. The Seedlings of the Atcrk5-1 Mutant Arabidopsis Have O_2_^•−^ and H_2_O_2_ Deficiencies in Root Tips

**Figure 1:**
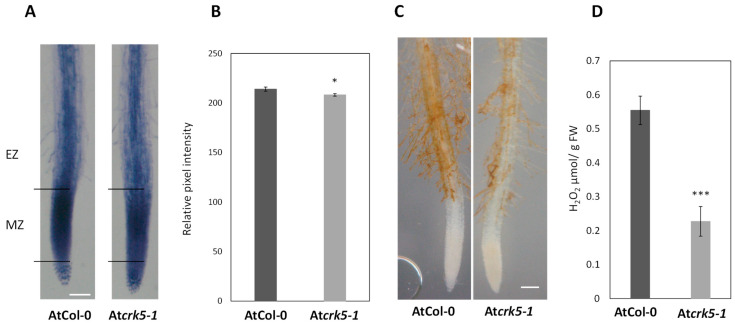
Histochemical staining of ROS in the *Arabidopsis* roots. (**A**) Superoxide anion staining by nitrosotetrazolium blue (NBT) in the wild type (AtCol-0) and the mutant (At*crk5-1*). Seedlings were grown vertically for 6 days. They were incubated for 5 min in NBT for staining. MZ = meristematic zone, EZ = elongation zone. Bar = 100 µm. (**B**) Quantification of the NBT content in the root tips of the wildtype and mutant seedlings was based on measuring the pixel intensity of equally sized areas of MZ by ImageJ. Asterisk indicates significant difference between the wild type and the mutant (Student’s *t*-test, * *p* < 0.1). (**C**) Hydrogen peroxide content of the wild type and mutant Arabidopsis roots stained by DAB (3,3′-diaminobenzidine). Seedlings were grown for 10 days then they were incubated in DAB. Representative pictures are shown for each line. Bar = 100 µm. (**D**) Hydrogen peroxide content (µmol per gram fresh weight) in wild type and mutant roots was measured spectrophotometrically. Asterisks indicate a significant difference between the wild type and the mutant (Student’s *t*-test, *** *p* < 0.001). Measurements were performed with at least 30 seedlings per each line. Bars indicate standard error. Three biologically independent experiments were carried out with the same statistical results.

ROS homeostasis basically influences root growth and the root gravitropic response [32,42,51,52]. Since the AtCRK5 protein was shown to be involved in the root gravitropic response [35], the distribution of O_2_^•−^ and H_2_O_2_ in the roots of the wild type (AtCol-0) and the mutant (At*crk5-1*) were detected by histochemical methods. When superoxide reacts with nitrosotetrazolium blue chloride (NBT), the immediate formation of formazan as blue precipitation can be visualized in root cells [53,54]. As shown in Figure 1A, NBT staining is confined mainly to the meristematic zone (MZ) of the wild type (AtCol-0) seedlings in agreement with earlier reports [53,54]. In the root tip of the At*crk5-1* mutant, the histochemical staining for superoxide anions (O_2_^•−^) was observed to be weaker. This was confirmed by the quantitative analysis of NBT staining (Figure 1B).

Since O_2_^•−^ is rapidly converted to H_2_O_2_, its distribution was also determined in the root tissues by using 3,3′-diaminobenzidine (DAB). H_2_O_2_ has been reported to preferentially accumulate in the differentiation zone of Arabidopsis roots [39,53]. Evaluation of the H_2_O_2_ content and distribution revealed differences between the wild type (AtCol-0) and the mutant (At*crk5-1*) root tips; the mutant root tips showed lower staining intensity with DAB in comparison to the wild type (Figure 1C). Quantitative measurement by Amplex Red confirmed that the A*tcrk5-1* mutant root tips contain much less H_2_O_2_ than the wild type ones (Figure 1D).

Taken together, there are O_2_^•−^ and H_2_O_2_ deficiencies in the At*crk5-1* root tips, which may contribute to the delayed gravitropic responses of this mutant.

### 2.2. Paraquat and H_2_O_2_ Treatments Restore the Gravitropic Response of Atcrk5-1 Roots

The herbicide paraquat (PQ) is widely used as a potent oxidative stress inducer [55]. It is well known that PQ is primarily reduced in chloroplasts capturing PSI electrons, but it is also reduced in mitochondria where complexes I and III are the electron donors [56]. In both cases, PQ can produce superoxide radicals (O_2_^•−^) formed from molecular oxygen.

**Figure 2:**
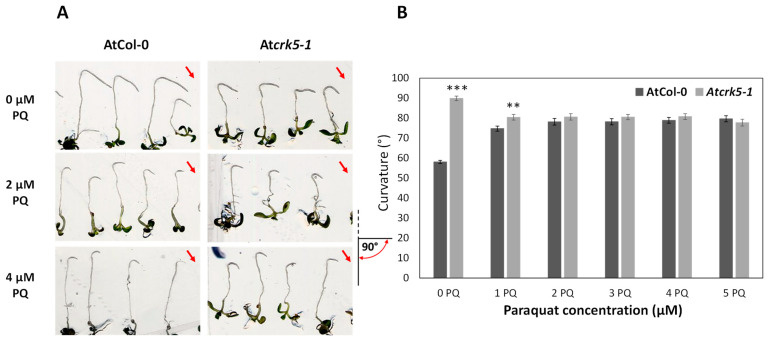
Effects of PQ on root gravitropic response. (**A**) Seedlings were grown vertically for 5 days, then they were transferred into media containing different PQ concentrations and were immediately rotated by −135°. The pictures were taken 24 h after gravistimulation. Red arrows indicate the direction of the gravity vector. The pictogram at the lower right corner of the Figure 1A shows the mode of the determination of the root curvature degree, the red semicircle with arrows indicates the measured angle (**B**) Quantitative analysis of PQ’s effect on the curvature of the gravistimulated roots. After 24 h of gravistimulation, the degrees of root bends were measured for each line grown at the various PQ concentrations (0–5 µM). Asterisks indicate significant differences between the wild type (AtCol-0) and mutant (At*crk5-1*) roots at ** *p* < 0.01 or at *** *p* < 0.001, respectively. Measurements were performed with at least 30 seedlings per each concentration. Bars indicate standard error. Three biologically independent experiments were carried out with the same statistical results.

To test the effect of PQ on the gravitropic response of Arabidopsis roots, vertically grown 5-days-old seedlings were put onto PQ-containing and PQ-free media and were immediately reoriented by −135° for 24 h, after which the degree of root bends were recorded. We found that PQ in the investigated concentration range (2–5 µM) had opposite effect on the gravitropic response of wild type and At*crk5-1* roots; the extent of root bending was decreased in the case of the wild type but increased in the mutant roots (Figure 2A,B). Interestingly, in the range of 2–5 µM, PQ restored the gravitropic response of the mutant to the wild type level (Figure 2A,B).

**Figure 3:**
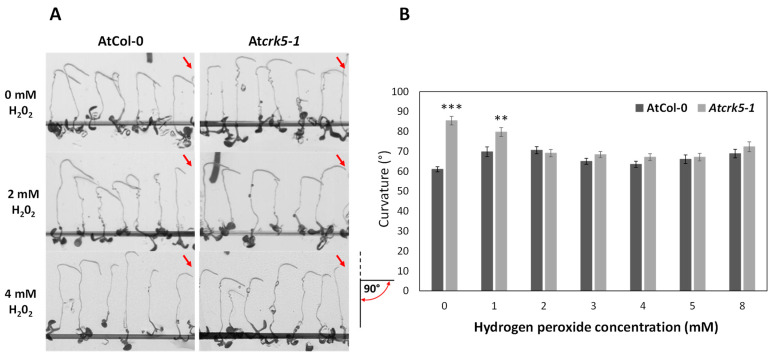
Effect of exogenous H_2_O_2_ on root gravitropic response. (**A**) Seedlings were grown vertically for 5 days, then they were transferred to media containing different H_2_O_2_ concentrations and immediately rotated by −135° for 24 h. Red arrows indicate the direction of the gravity vector. The pictogram at the lower right corner shows the mode of the determination of the root curvature degree, the red semicircle with arrows indicates the measured angle. (**B**) Quantitative analysis of the H_2_O_2_ effect on the degree of gravitropic curvatures of wild type (AtCol-0) and mutant (At*crk5-1*) roots. Asterisks indicate significant differences at ** *p* < 0.01 or *** *p* < 0.001 between the wild type (Col-0) and the mutant (At*crk5-1*). Measurements were performed with at least 30 seedlings per each line. Bars indicate standard error. At least three biologically independent experiments were carried out with the same statistical results.

We also investigated the effect of exogenous H_2_O_2_ applied at 0–8 mMconcentrations for 24 h on the gravitropic response of roots (Figure 3A,B). It affected the gravitropic response of the wild type and At*crk5-1* mutant roots similarly to PQ (Figure 2A,B). Significant differences were found in root bends between the wild type (AtCol-0) and mutant (At*crk5-1*) seedlings in the absence and at the lowest (1 mM) exogenous H_2_O_2_ concentration (Figure 3B). This difference, however, disappeared at higher doses of H_2_O_2_ (2–8 mM) (Figure 3B).

These data showed that exogenous PQ as well as H_2_O_2_ were able to ameliorate the gravitropic response of the At*crk5-1* mutant roots indicating a potential role for the kinase in the maintenance of ROS homeostasis at the root tip.

### 2.3. PQ or H_2_O_2_ Treatments Restore the Auxin Distribution in the Root Meristem of the Atcrk5-1 Mutant during Gravistimulation

**Figure 4:**
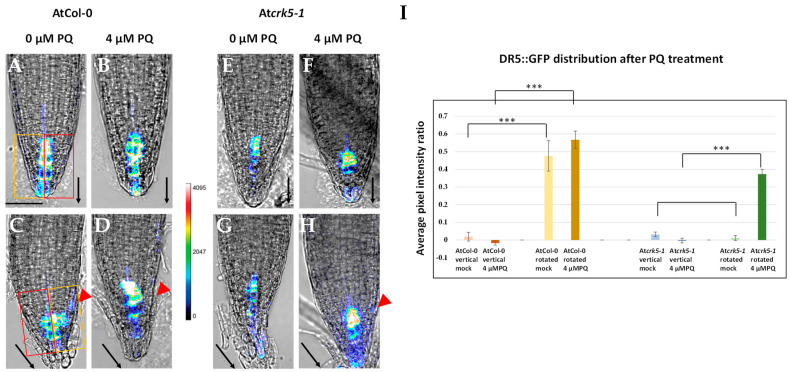
Fluorescence intensity heat maps of DR5::GFP signals in root tips. Activity of the auxin-induced DR5::GFP reporter in the 6-day-old wild type (AtCol-0; **A**–**D**) and mutant (At*crk5-1*; **E**–**H**) roots during vertical growth (**A**,**B**,**E**,**F**) or after gravistimulation (−135° rotation for 4–5 h; **C**,**D**,**G**,**H**) in the absence (**C**,**G**) and presence (**D**,**H**) of 4 µM PQ. (**I**) Quantification of the DR5::GFP fluorescence intensity ratio by investigating the average pixel intensities of the GFP signals measured in equally-sized areas at both sides of the root by ImageJ/Fiji. At least 5–10 images from the wild type and the mutant categories were analysed in each version from three independent experiments. Values near zero represent a symmetrical DR5::GFP signal position, while values higher than 0 mean an asymmetrical DR5::GFP signal position. Asterisks indicate significant difference between the corresponding mock control and the treatment (two-way ANOVA, means comparison was carried out by Bonferroni; *** *p* < 0.001). The fluorescence intensity was translated into a colour code (scale is in the middle). Black arrows show the direction of the gravity vector. Red arrowheads indicate the lateral redistribution of the signal towards the gravity vector. Rectangles on (**A**,**C**) images represent the equally sized areas. Scale bar: 50 µm. The original DR5::GFP fluorescence images (without heatmap) are shown as Supplemental Figure S1.

Redistribution of auxin plays an important role in plant gravitropism [1,11,57,58,59,60,61]. We followed the establishment of the auxin gradient by the auxin induced DR5::GFP construct [59] in vertically placed and rotated roots with and without PQ treatment. Six-day-old seedlings were transferred into the control and the 4 µM PQ-containing media, respectively, and half of the petri dishes were kept vertically while the others were immediately rotated by −135°. The effect of PQ on the DR5::GFP signal was checked 4–5 h after rotation by confocal laser scanning microscopy (CLSM; Figure 4).

When the AtCol-0 roots grew vertically, there was a symmetrical DR5::GFP signal in the quiescent centre and the columella (Figure 4A), which was not significantly affected by the application of 4 µM PQ for 4–5 h (Figure 4B). Due to the gravistimulation of wild type roots (−135° rotation for 4–5 h), the DR5::GFP signal started to be asymmetric in the absence of PQ (Figure 4C), which could also be observed in the presence of 4 µM PQ and the signal was also intensified (Figure 4D). Under normal conditions (at vertical position, 0 µM PQ), the At*crk5-1* mutant showed much less intense DR5::GFP signal in the root meristem as compared to the wild type (Figure 4E). The addition of 4 µM PQ to the medium did not significantly alter the intensity or distribution of this DR5::GFP signal (Figure 4F). Upon gravistimulation, the DR5::GFP signal remained symmetrical in the At*crk5-1* mutant root tip (Figure 4G) in contrast to that of the wild type control, as was previously reported by [35]. Surprisingly, the addition of 4 µM PQ to the At*crk5-1* roots resulted in asymmetry in an intensified DR5::GFP signal under gravistimulation (Figure 4H). Quantification of the DR5::GFP signal symmetry/asymmetry is shown in Figure 4I. Statistical analysis of the values confirmed the effect of PQ on the asymmetry in auxin distribution.

Our data suggest that exogenous paraquat (oxidative stress) restores the capability of the otherwise gravitropically impaired At*crk5-1* roots for bending via affecting the redistribution of auxin in the root meristem.

**Figure 5:**
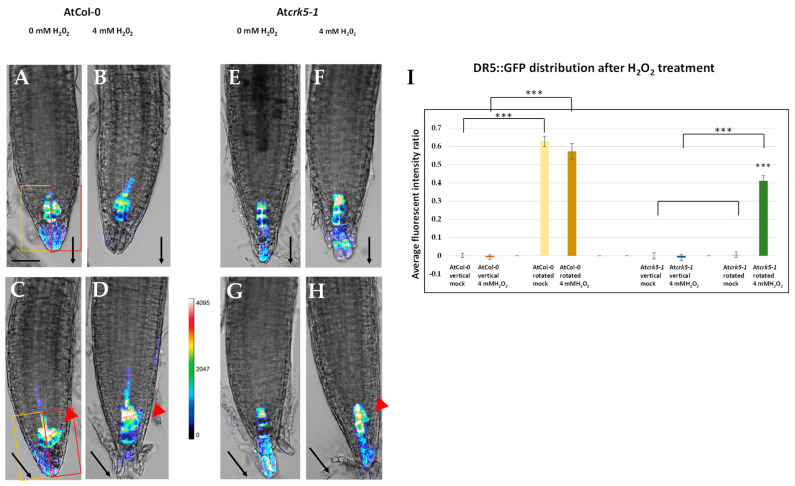
H_2_O_2_ treatment restores the distribution of the DR5::GFP signal in gravistimulated At*crk5-1* root meristems. Distribution of the DR5::GFP signal in the 6-day-old AtCol-0 wild type (**A**–**D**) and mutant At*crk5-1* (**E**–**H**) seedling roots without (**A**,**C**,**E**,**G**) or treated with 4 mM H_2_O_2_ (**B**,**D**,**F**,**H**). Vertically-grown (**A**,**B**,**E**,**F**) and gravistimulated (−135° rotation for 4–5 h; **C**,**D**,**G**,**H**) roots were compared. (**I**) Quantification of the DR5::GFP fluorescence intensity ratio by investigating the average pixel intensities measured in equally sized areas at both sides of the GFP signals by ImageJ/Fiji. At least 5–10 images from the wild type and mutant categories were analysed in each version from three independent experiments. Values near zero represent a symmetrical DR5::GFP signal position, while values higher than 0 mean an asymmetrical DR5::GFP signal position. Asterisks indicate significant differences between the corresponding mock control and the treatment (two-way ANOVA, means comparisons was carried out by Bonferroni; *** *p* < 0.001). The fluorescence intensity was translated into a colour code (scale is in the middle). Black arrows show the direction of the gravity vector. Red arrowheads indicate the lateral redistribution of auxin towards the gravity vector. Rectangles on (**A**,**C**) images represent the equally sized areas. Scale bars = 50 µm. The original DR5::GFP fluorescent images (without heatmap) are shown as Supplemental Figure S2.

In addition to that of PQ, the effect of exogenous H_2_O_2_ (4 mM) on the distribution of the DR5::GFP signal was investigated during the gravitropic response (Figure 5.). The experiment was carried out in the same way as with the PQ. Figure 5 shows the distribution of the DR5::GFP signal during the graviresponse of the wild type (AtCol-0) and the mutant (At*crk5-1*) seedling root tips with and without 4 mM H_2_O_2_ in the medium. Similarly to PQ, exogenous H_2_O_2_ restored the asymmetric distribution of the DR5::GFP signal in the root meristem of the At*crk5-1* mutant (Figure 4 and Figure 5). Quantification of the DR5::GFP signal symmetry/asymmetry is shown in Figure 5I. Statistical analysis supported the view that the effect of H_2_O_2_ on the auxin distribution in gravistimulated mutant roots is similar to that of the PQ treatment.

### 2.4. PQ Restores the PIN2-GFP Distribution in the Root Meristem of the Atcrk5-1 Mutant during Gravistimulation

**Figure 6:**
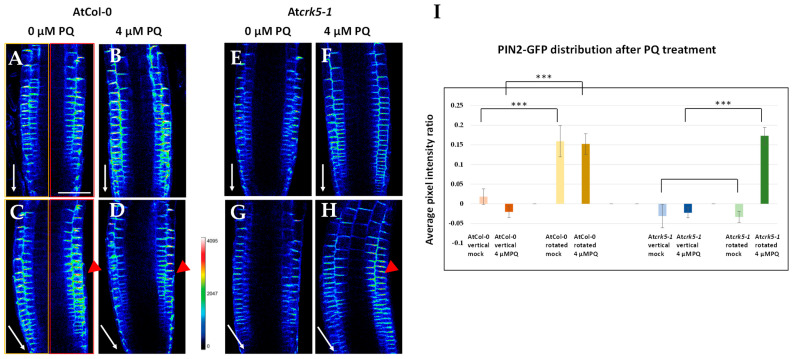
PQ treatment restores the distribution of the PIN2-GFP signal in At*crk5-1* Arabidopsis root meristems during gravistimulation. Distribution of the PIN2-GFP signal in 6-days-old AtCol-0 wild type (**A**–**D**) and mutant At*crk5-1* (**E**–**H**) seedling roots without (**A**,**C**,**E**,**G**) or treated with 4 μM PQ (**B**,**D**,**F**,**H**). Vertically-grown (**A**,**B**,**E**,**F**) and gravistimulated (−135° rotation for 4–5 h; **C**,**D**,**G**,**H**) roots were compared. (**I**) Quantification of the PIN2-GFP fluorescence intensity ratio by investigating the average pixel intensities measured in equally sized areas at both sides of the GFP signals by ImageJ/Fiji. At least 5–10 images from wild type and mutant categories were analysed in each version from three independent experiments. Values near zero represent a symmetrical DR5::GFP signal position, while values higher than 0 mean an asymmetrical PIN2-GFP signal position. Asterisks indicate significant difference between the corresponding mock control and the treatment (two-way ANOVA, means comparisons was carried out by Bonferroni; *** *p* < 0.001). The fluorescence intensity was translated into a colour code (scale is in the middle). White arrows show the direction of the gravity vector. Red arrowheads indicate the lateral redistribution of the PIN2-GFP signal towards the gravity vector. Rectangles on (**A**,**C**) images represent the equally sized areas. Scale bar: 50 µm. The original fluorescent images (without heatmap) are shown as Figure S3.

The auxin efflux protein PIN2 is responsible for the basipetal auxin transport in Arabidopsis roots and thus contributes to the regulation of the gravitropic response [10,11,13,19,35]. Localization of this polar auxin transporter was reported to be changed in the At*crk5-1* mutant in relation with the delayed gravitropic responses of its roots [35]. Since it was found that PQ or H_2_O_2_ treatments restored the auxin distribution and the gravitropic bending of the At*crk5-1* roots (Figure 2, Figure 3 and Figure 4), we investigated the localization of the PIN2-GFP signal in the transition zones (TZ) of the wild type and At*crk5-1* mutant roots in response to PQ (4 µM) treatment during gravistimulation (Figure 6).

The vertically positioned AtCol-0 roots showed symmetrical PIN2-GFP signal distribution in the epidermis and cortex cell layers of roots (Figure 6A). This symmetrical localization was not affected by adding 4 µM PQ, however, the signals became stronger indicating somewhat higher PIN2 protein levels (Figure 6B). During the gravistimulation of the wild type roots, the PIN2-GFP signal became asymmetric in what was not altered by the presence of 4 µM PQ in the medium (Figure 6C,D). The roots of the At*crk5-1* mutant growing in vertical position had a somewhat fainter symmetrical PIN2-GFP signal (Figure 6E). The addition of 4 µM PQ to the medium did not alter the distribution of the PIN2-GFP signal in the mutant roots either, but the intensity of the signal was also increased (Figure 6F). Upon gravistimulation, the PIN2-GFP signal remained symmetrical in the At*crk5-1* mutant root tip (Figure 6G) as it has been previously described [35]. However, the presence of 4 µM PQ resulted in the asymmetric distribution of the PIN2-GFP signal in the gravistimulated At*crk5-1* roots (Figure 6H) Quantification of the PIN2-GFP signal symmetry/asymmetry is shown in Figure 5I. The results of the quantitative analysis supported the idea that PQ treatment rescued the gravitropic bending ability of the mutant roots via positively influencing the asymmetric PIN2-GFP signal formation.

The above described results indicate that the crosstalk between ROS (the PQ generated O_2_^•−^ and H_2_O_2_) and auxin transport via the regulation of PIN2 protein distribution is disturbed by the At*crk5-1* mutation.

### 2.5. Increased Nitric Oxide Level in Its Root Apices Contributes to the Delayed Gravitropic Bending of the Atcrk5-1 Mutant

**Figure 7:**
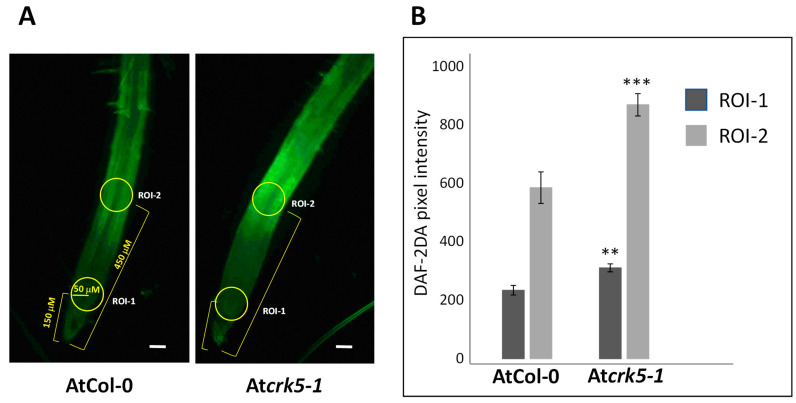
Detection of NO in wild type and mutant Arabidopsis root apexes. (**A**) Evaluation of NO content by the fluorescence probe DAF-FM-DA (4-amino-5-methylamino-2′,7′-difluorofluorescein diacetate) in wild type (AtCol-0) and mutant (At*crk5-1*) root apices. Yellow circles represent the ROI-1 and ROI-2 (Region of Interest) in the studied roots. Scale bar = 50 µm. (**B**) Quantification of the DAF-FM DA-mediated fluorescence signal in wild type and mutant Arabidopsis root apices. DAF-FM fluorescence was measured at two identical circular regions 150 µm (dark grey, ROI-1) and 450 µm (light grey, ROI-2) away from the root tips as shown in (**A**) by yellow circles. Means (±SE) were analysed from three biological repeats (*n* ≥ 25). *p*-values were calculated with two-tailed Student’s *t*-test, ** *p* ≤ 0.01, *** *p* ≤ 0.001.

In addition to ROS, NO is also known to affect the gravitropic response of roots [29,30,62]. Therefore, the NO level and distribution were also determined in the wild type and At*crk5-1* roots. The cell-permeable, NO-sensitive fluorophore DAF-FM-DA (4-amino-5-methylamino-2′,7′-difluorofluorescein diacetate) was used for this purpose. NO is known to accumulate at the meristematic zone (MZ), transient zone (TZ), and elongation zone (EZ) of Arabidopsis roots [50]. We observed that in the At*crk5-1* mutant roots there was higher DAF-FM fluorescence signal in the meristematic and elongation zones in comparison to the wild type ones (Figure 7A). Quantification of the fluorescence emission data confirmed that this difference is statistically significant (Figure 7B).

**Figure 8:**
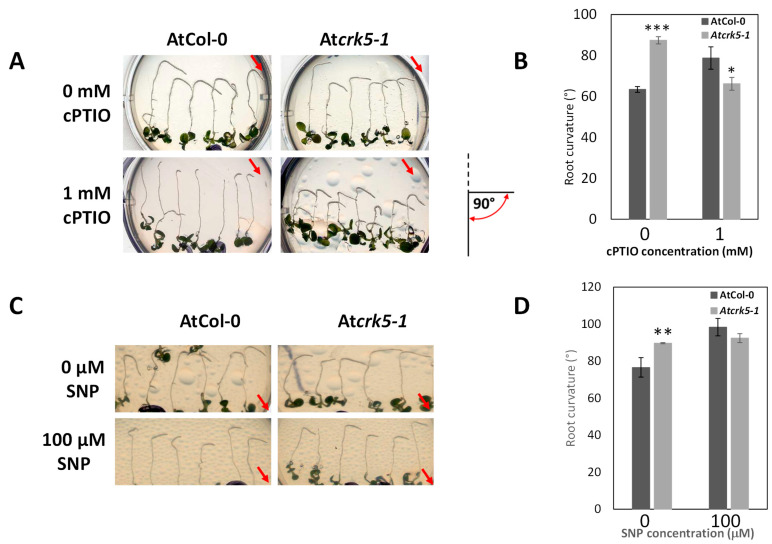
Effect of the manipulation of the endogenous NO level on root gravitropic response. Seedlings were grown vertically for 5 days, then they were transferred to media containing the NO scavenger cPTIO (2-4-carboxyphenyl-4,4,5,5-tetramethylimidazoline-1-oxyl-3-oxide, **A**,**B**) or the NO donor SNP (sodium nitroprusside, **C**,**D**) and immediately rotated by −135° for 24 h. Representative pictures (**A**,**C**) and quantitative analysis of the effect on the gravitropic bending of the wild type (AtCol-0) and mutant (At*crk5-1*) roots (**B**,**D**) are shown. Red arrows indicate the direction of the gravity vector. The pictogram at the Figure 8**A** shows the mode of the determination of the root curvature degree, the red semicircle with arrows indicates the measured angle. Asterisks indicate significant differences at * *p* < 0.1, ** *p* < 0.01 and *** *p* < 0.001 between wild type (AtCol-0) and mutant (At*crk5-1*). Measurements were performed with at least 30 seedlings per each line. Bars indicate standard error. At least two biologically independent experiments were carried out with the similar statistical results.

To confirm the significance of NO in the delayed gravitropic response of the mutant, wild type and mutant roots were treated with the NO scavenger cPTIO (2-4-carboxyphenyl-4,4,5,5-tetramethylimidazoline-1-oxyl-3-oxide, 1 mM) or the NO donor SNP (sodium nitroprusside, 100 µM) and their gravitropic bending was determined after 24 h of gravistimulation. Both cPTIO (Figure 8A,B) and SNP (Figure 8C,D) decreased the gravitropic bend of wild type roots confirming the view that too high or too low endogenous NO levels equally impair the gravitropic response of Arabidopsis roots [30]. However, when the NO scavenger cPTIO (1 mM) was applied to the gravistimulated At*crk5-1* mutant roots, it enhanced their graviresponse (Figure 8A,B), supporting the view that there is a link between the elevated NO level and the impaired gravitropic curvature of the At*crk5-1* mutant roots. The NO donor SNP had no significant effect on the gravitropic band of the mutant roots, indicating that the NO level in these roots was high enough to prevent the gravitropic response even without SNP (Figure 8C,D).

### 2.6. PQ Treatment Restores the Wild Type NO Level in Atcrk5-1 Mutant Root Tips

**Figure 9:**
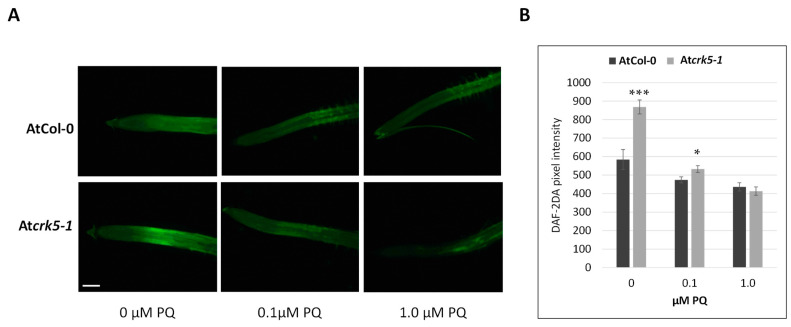
Detection of NO in wild type and mutant vertically grown Arabidopsis root apices without and with PQ treatment (0.1 or 1 µM). NO content was measured by the fluorescence probe DAF-FM-DA (4-amino-5-methylamino-2′,7′-difluorofluorescein diacetate) (**A**, scale bar: 100 µm) and expressed as pixel intensity (**B**). DAF-FM fluorescence was measured at two identical regions, cc. 200 µm and 500 µm far away from the root tips and averaged (see Figure 7). See that 1 µM PQ concentration reduced the NO content of the At*crk5-1* mutant to the wild type level. Bars indicate ±SE of measurements performed with at least 20 seedlings per each line. *p*-values were calculated with two-tailed Student’s *t*-test (* *p* ≤ 0.1; *** *p* ≤ 0.001). Two biologically independent experiments were carried out with similar statistical results.

It is well demonstrated that there is a crosstalk of ROS and NO in many developmental processes [63], including the establishment of root architecture [49]. We tested, therefore, whether this crosstalk operates in the At*crk5-1* mutant roots. It was found that under increasing PQ concentration, the NO content of the mutant roots was reduced to the level of the wild type ones. Considering the observations that lowering the NO level to that of the wild type in the At*crk5-1* roots restored the gravitropic response of the mutant (Figure 8A,B) similar to the PQ treatment (Figure 2), and that PQ reduced the NO level in the mutant roots (Figure 9), one may suppose that ROS exert their effect on the gravitropic response of the At*crk5-1* roots via controlling NO accumulation.

## 3. Discussion

### 3.1. The AtCRK5 Kinase Controls the Root Gravitropic Response Facilitating the Redistribution of Auxin

The AtCRK5 protein kinase is one of the members of the Ca^2+^/Calmodulin-Dependent Protein Kinase-Related Kinases (CRKs) subfamily. We have previously reported that this kinase has a direct role in the regulation of root gravitropic response [35].

It is well accepted that the bending of the root towards the gravity vector is due to the asymmetry in the auxin distribution between the lower and upper tissues of the horizontally oriented root tip [1,57,58,59,61,64]. The asymmetry in auxin distribution is due to the gravity-induced differential regulation of the turnover of PM-bound auxin transporter proteins (including AUX1, PIN3, and PIN2) at the two sides of the root. The central role of the PIN2 auxin efflux carrier in the gravitropic response of the root is especially well established. Changing the direction of gravity PIN2 transiently accumulates at the basal cell membranes in the epidermis at the lower side of the root tip transition zone [11,15,16]. Auxin transported at higher quantity at this side differentially inhibits cell elongation that results in the downward turning of the growing root.

AtCRK5 is membrane associated due to its N-terminal myristoylation/palmitoylation motif, and its membrane localisation pattern in the root partially overlaps with that of PIN2 [35,65]. Moreover, the AtCRK5 kinase was shown to in vitro phosphorylate the hydrophilic lop of PIN2 [35]. It is of note that the AtCRK5 protein kinase also has role in the negative gravitropism of the shoot [34,35] and can also phosphorylate the auxin efflux transporter PIN3 affecting hypocotyl bending in skotomorphogenesis [37] and the PIN1, PIN4, and PIN7 auxin efflux transporters acting in embryogenesis [38], indicating its general role in the fine tuning the auxin transport during plant development.

It is well established that the phosphorylation state of hydrophilic T-loop residues of the PIN2 controls whether it is stabilized in the basal cell membrane or goes through apical transcytosis [19,66]. In the gravistimulated At*crk5-1* mutant root tip, PIN2 was found to be depleted from the apical membranes of epidermal cells due to accelerated brefeldin-sensitive internalization and showed either apolar or apical localization in the neighbouring cortical cells due to transcytosis [35]. In addition, the expression level of the auxin marker DR5::GFP was lower at the root tip area of the mutant than that of the wild type [35]. Based on these experimental observations, it is hypothesized that the At*crk5-1* mutation enhances PIN2-mediated shootward auxin flow from the root tip through the cortex toward the elongation zone and in this way depletes auxin from the root meristem and interferes with its gravitropic redistribution [35,60].

Here, we provided further experimental evidence that the absence of the AtCRK5 protein kinase delays the gravitropic response of the roots in correlation with low PIN2 abundance in the root transition zone, limited auxin accumulation in the meristematic region, and inhibited redistribution of PIN2 as well as auxin in response to the gravitropic stimulus (Figure 4, Figure 5 and Figure 6A,C,E,G). Interestingly, we found that exogenous application of H_2_O_2_ or the O_2_^•−^ (and subsequently H_2_O_2_) generating PQ could rescue the gravitropic response of the At*crk5-1* mutant (Figure 1, Figure 2, Figure 4, Figure 5 and Figure 6B,D,F,H). These observations raised the question how these reactive oxygen species fit into the above model explaining the contribution of the AtCRK5 kinase to the root gravitropic response.

### 3.2. AtCRK5 Is Required to Maintain the ROS Homeostasis at the Root Tip Region That Is Needed for Proper Root Growth and Gravitropic Response

Staining Arabidopsis root tips with specific ROS-sensitive dyes revealed that the At*crk5-1* mutant has lower superoxide anion (O_2_^•−^) and hydrogen peroxide (H_2_O_2_) contents in the root tip region than the wild type (Figure 1). This is in agreement with the restriction of root growth by app. 30% in the mutant in comparison to the wild type [35], since it is generally accepted that ROS play important roles in the control of root growth and development [39]. The accumulation sites of the two ROS, superoxide radical anion and hydrogen peroxide, are different in Arabidopsis root tips: O_2_^•−^ can be preferentially detected in the meristematic/elongation zone, whereas H_2_O_2_ rather accumulates in the differentiation zone [53,67]. Moreover, they were reported to have the opposite effect on root elongation: decreasing O_2_^•−^ concentration reduced root elongation, while removing H_2_O_2_ by scavengers promoted it [53]. These two ROS are considered to control the transition between cell proliferation and differentiation in RAM [39,52,53]. The superoxide anion (O_2_^•−^) is known to control cell proliferation in the root apex region [39,52,54]. H_2_O_2_ generated in the apoplast can be converted into more reactive free radicals, e.g., hydroxyl radicals (^•^OH) controlling cell elongation. The ^•^OH radical facilitates cell wall loosening [39]. Liszkay et al. 2004 [40] revealed that root elongation growth is a function of cell wall peroxidase generated ^•^OH radicals in maize. The formation of ^•^OH radicals was shown to be dependent on the PM-localized NAD(P)H oxidase, catalysing the production of O_2_^•−^ that was subsequently converted to H_2_O_2_. ROS might also influence root growth affecting microtubule organisation [68], microtubule-related PIN2-recycling [42,69], interfering with auxin redistribution [33], and attenuating the auxin signal transduction [41].

Besides root growth, the root gravitropic response also essentially depends on ROS homeostasis [31,32,33]. It was reported that ROS, presumably H_2_O_2_, differentially modulate root tropic responses, promoting gravitropism, but negatively regulating hydrotropism [32]. The promotion of gravitropism by ROS, including their production, was found to be auxin-dependent [31] and involved the activation of a major membrane-bound NADPH oxidase via a PI3K-dependent pathway [25]. These data put ROS/H_2_O_2_ downstream of auxin in the gravitropic response. Therefore, the altered ROS level in the At*crk5-1* mutant roots might either directly contribute to the observed root growth and gravitropic defects or the reduced auxin content of the mutant root tip prevents ROS/H_2_O_2_ production contributing to the delayed gravitropic response.

Our results rather support the view that ROS produced at the root tip are upstream of auxin in the control of the gravitropic response, especially as auxin transport is considered. Either PQ or H_2_O_2_ treatment could rescue the gravitropic response of the At*crk5-1* mutant roots in correlation with the restored abundance and asymmetric distribution of the PIN2 auxin efflux carrier at the root tip transition zone. In consequence, the asymmetric distribution of auxin was also restored, thus evoking the gravitropic bending of the root. This is in agreement with the observations of Zhou et al. [33] who reported that exogenous hydrogen peroxide inhibited gravitropism due to the inhibition of auxin redistribution. It is of note that in our experiments, either PQ or H_2_O_2_ at the applied concentration slightly reduced the gravitropic curvature of the AtCol-0 wild type roots, indicating that endogenous ROS has to be within an optimal concentration range for the proper gravitropic response.

Our results, however, seems to be contradictory to those of Joo and co-workers [25,31], who experimentally proved that ROS are involved in root gravitropism downstream of auxin signalling. Auxin-induced ROS were hypothesized to trigger the activation of the mitogen-activated protein kinase signalling [31] but the involvement of apoplastic ROS in cell wall remodelling during the gravitropic response cannot be excluded either [52]. ROS has been found to be in a feed-forward loop with auxin signalling during lateral root formation via the formation of lipid peroxide-derived reactive carbonyl species [51]. The operation of similar pathways might also be envisaged during primary root growth and gravitropism.

The seeming contradiction in the role of ROS upstream as well as downstream of auxin during gravitropism can be elucidated taking into consideration the positive feedback auxin exerts on its own transport. Auxin is known to promote the expression, membrane targeting, and stability of PIN efflux carriers, and this positive feedback operates during the gravitropic response as well [11,13,15,70]. Even if the primary target of the AtCRK5 kinase is PIN2, and its absence primarily compromises auxin transport and signalling, then the auxin-induced ROS level needs to be low in the At*crk5-1* mutant, as we observed. The reduced ROS level may further interfere with PIN2 membrane targeting that strengthens the negative feedback. In this scenario, PQ/H_2_O_2_ application might stabilize PIN2 in the plasma membrane of cortical root cells of the At*crk5-1* mutant independent of its phosphorylated state [35] that restores the auxin level at the root tip available for redistribution during the gravitropic response. The increased auxin level has a positive feedback on PIN2 turnover, stability, and targeting. The fact that applying PQ generating intracellular O_2_^•−^ (that might be promptly converted to H_2_O_2_), or exogenous H_2_O_2_ could rescue the PIN2 turnover, auxin redistribution, and gravitropic defects of the At*crk5-1* mutant indicates that the limited ROS level is central in the gravitropic phenotype of the mutant.

Although the AtCRK5 kinase was shown to be capable of phosphorylating the hydrophilic T-loop of various PIN auxin efflux carriers including PIN2 supporting the above model [35,37,38], its role in the direct regulation of ROS levels cannot be excluded either. In this second scenario, the absence of the AtCRK5 kinase directly inhibits ROS generation (or enhances their removal) that negatively affects PIN2 turnover/stability and consequently the auxin transport resulting in the observed phenotypes. According to both scenarios, ROS must have positive effect on PIN2 turnover and auxin transport. Although prolonged exposition of roots to high levels of H_2_O_2_ was shown to negatively affect the endocytic recycling of PIN2, its membrane abundance, and gravitropism [33,42], one can hypothesize that ROS within a lover concentration range optimal for root growth might be required for proper auxin transport. Our results indicate that this positive effect of ROS might be indirect and involves NO.

### 3.3. Nitric Oxide Might Mediate the Effect of AtCRK5-Dependent ROS Generation on Auxin Transport

In addition to ROS, auxin also has an intricate relationship with NO controlling root development. NO may act either downstream or upstream of auxin signalling during adventitious root, lateral root, or root hair formation as well as primary root growth [43,44,45]. In these auxin-dependent processes, NO may serve as a second messenger in auxin signal transduction pathways [46,71,72,73]. However, NO was also reported to positively control auxin signalling via S-nitrosation of the TIR1/ABF auxin receptor [48] but negatively control PIN-mediated auxin transport [47]. Either the depletion [46] or overproduction of NO [47] interfered with the primary root growth, reducing meristem size and cell division activity. These observations indicated that, similarly to ROS, an optimal NO concentration range is required at the root tip to maintain the root meristem function and growth.

Auxin-induced asymmetric NO accumulation was shown to modulate the gravitropic bending in soybean roots [29]. The importance of the NO-mediated regulation of PIN2 trafficking during the gravitropic responses of Arabidopsis roots was also demonstrated by [30]. In both species, upon gravistimulation NO transiently exhibited an asymmetric distribution between the lower and upper sides of the root, with higher levels towards the gravitropic vector. Decreasing the NO level by the NO scavenger cPTIO resulted in the inhibition of the asymmetric distribution of both NO and PIN2, and consequently, the gravitropic bending. Since increased NO levels were reported to reduce PIN1 abundance at root meristems [47] and decreased NO levels negatively influenced PIN2 distribution [30], both resulting in reduced graviresponses of roots, it was speculated that NO may specifically modulate the endocycling, trafficking, and degradation of the different PIN proteins in Arabidopsis root cells [30]. This hypothesis is supported by our recent findings that NO can S-nitrosate the ROP2 GTPase implicated in the control of the endocytic recycling of PIN proteins and that loss of ROP2 function suggests Arabidopsis mutants are insensitive to the root growth inhibiting effect of NO [74].

It was found that the At*crk5-1* mutant has higher NO levels at the transition zone of its root meristem than the wild type (Figure 7). This is in contrast to the ROS, whose levels were reduced (Figure 1). Increasing the ROS level using PQ restored the NO level in the At*crk5-1* mutant to that of the wild type, allowing for a proper gravitropic response (Figure 9). Using the NO-scavenger cPTIO could also rescue the gravitropic bending of the mutant roots (Figure 8). Since cPTIO-mediated root growth inhibition cannot be seen in the At*crk5-1* mutant (Figure 8A), one can suppose that cPTIO returns the higher endogenous NO level of the mutant to normal, allowing for proper growth and gravitropism. Interestingly, the depletion of NO was correlated with elevated levels of ROS in Arabidopsis root [46]. Among others, the auxin-dependent S-nitrosation of cytosolic ascorbate peroxidase (cAPX) is one of the means regulating the H_2_O_2_ level in plant roots [44]. Altogether, these observations indicate that ROS and NO are in an intricate relationship with each other and regulate each other’s homeostasis that is required for normal root growth and geotropism. PQ-generated O_2_^•−^ and H_2_O_2_ seem to positively regulate NO production while ROS downregulate the NO level during the regulation of gravitropic bending. The complex interplay of ROS and NO controlling each other’s homeostasis is a general theme in plant physiology and development [44,63,75,76,77]. It is also well accepted that auxin has an upstream role regulating the ROS and NO interplay in plant roots [43,44].

### 3.4. The AtCRK5 Kinase Fine Tunes the Feedback Loop Including PIN2, Auxin, ROS, and NO That Controls the Gravitropic Response of Arabidopsis Roots

**Figure 10:**
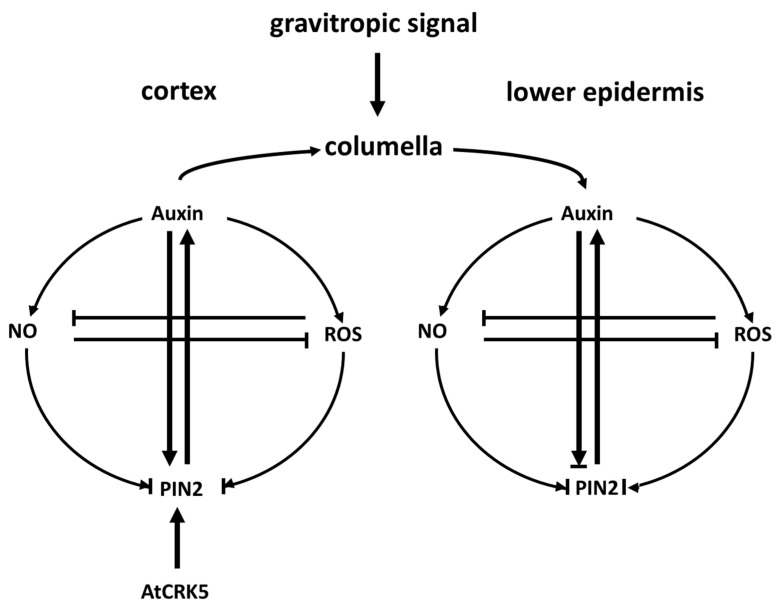
Hypothetical model integrating the current knowledge about the role of auxin, NO, ROS, and AtCRK5 kinase in the root gravitropic response in Arabidopsis. Arrowheads indicate positive, blunt ends indicate negative regulation. Note that some of the interactions are only transient. Note that the effect of ROS, NO, and auxin on PIN2 endocytosis/turnover can be positive and negative depending on concentration and timing. For a detailed description see the text. NO—nitric oxide; ROS—reactive oxygen species; PIN2—PINOID 2 auxin efflux carrier; AtCRK5—*Arabidopsis thaliana* Calcium-Dependent Protein Kinase-Related Kinase 5.

Since both ROS and NO production and asymmetric redistribution is induced by auxin during the gravitropic response of roots [29,31,63], and both act on PIN2 turnover and auxin transport [30,42] (Figure 6), a regulatory feedback loop involving auxin, ROS, and NO operating during the early gravitropic response of roots can be envisioned (Figure 10). Since the only currently known potential substrates of AtCRK5 are the PIN proteins, our current model is that AtCRK5 contributes to this feedback regulation during the gravitropic response via phosphorylating PIN2 in the cortical cells (Figure 10). This phosphorylation is required to stabilize the PIN2 protein in the apical membrane of root cortex cells [35] that is needed for a correct gravitropic response [60]. In this way, sufficient auxin is transported to the meristem that gets redistributed asymmetrically upon gravistimulation [58,59,61]. The asymmetric auxin induces asymmetric ROS as well as NO accumulation at the lower epidermis of the root [30,32]. ROS and NO transiently inhibits PIN2 internalisation strengthening shootward auxin transport that further increases PIN2 stability [30,33,42]. Since the accumulation of both ROS and NO in response to the gravistimulation is transient, following their induction by auxin, they probably return to basal levels due to their cross regulation [63]. It was shown that the effect of auxin on PIN2 stability is time dependent. Short auxin treatment inhibits PIN2 endocytosis and interferes with the gravitropic response while a longer treatment speed up the turnover of the protein [11]. The transient auxin-induced accumulation of ROS and NO may contribute to the short-term effect of auxin. In the next stage, the self-regulation of auxin transport is sufficient to maintain the auxin gradient [13] at the root tip, controlling differential cell elongation and root bending. When auxin in the lower epidermic cells reaches a threshold level, auxin start to promote PIN2 protein turnover to prevent further bending [11].

Our results presented in this manuscript support the view that the absence of AtCRK5 activity results in increased PIN2 turnover in the root cortex cells, limited acropetal auxin transport and therefore interferes with auxin accumulation at the root tip that unbalances the ROS-NO homeostasis. That is why the ROS level is decreased and the NO level is augmented in the transition zone. The limited auxin and the unbalanced ROS and NO levels prevent the asymmetric redistribution of these compounds that limits the differential stabilization of PIN2 in the lower epidermal cells and thus the positive self-regulation of the asymmetric auxin transport is not established. Lowering the NO and increasing the ROS levels to normal seems to be sufficient to prevent the negative feedback of the reduced auxin accumulation on PIN2 stability at the root tip (Figure 6). The application of PQ or H_2_O_2_ therefore allow for the building up of the self-regulating auxin gradient between the upper and lower epidermal cells, even in the absence of AtCRK5 function at the cortex allowing the root bending.

## 4. Materials and Methods

### 4.1. Plant Material and Growth Conditions

All plants used in this study are in *Arabidopsis thaliana* (L.) Columbia-0 ecotype (AtCol-0) background. Identification of the At*crk5-1* (MPIZ38225) mutant, assembly of the auxin sensor DR5::GFP, and the PIN2-GFP auxin efflux gene constructs in *Agrobacterium tumefaciens* binary vectors were described in [35]. All seeds were propagated in the greenhouse facility of the Eötvös Loránd Research Network, in the Biological Research Centre, Institute of Plant Biology, Director: Prof. Imre Vass (ELKH-BRC).

In general, wild type and mutant seeds were surface sterilized and kept at 4 °C for two days. The imbibed seeds were transferred onto plates containing half-strength Murashige and Skoog medium (MS) [78] with 0.5% sugar (Molar Chemicals Kft, Budapest, Hungary), 0.8% phytoagar (Duchefa Biochemie, Haarlem, The Netherlands), pH: 5.7, and germinated under continuous light condition (50 µmole photons m^−2^ s ^−1^ light intensity, 22 °C) [35] in the ELKH-BRC plant growth chambers.

### 4.2. Root Gravitropic Assays

Seeds of wild type *Arabidopsis thaliana* (Columbia-0) and mutant At*crk5-1* line were germinated after two days of stratification at 4 °C on 1/2-strength MS medium containing 0.5% sucrose in a growth chamber as described in [35]. The plates were placed vertically for root gravitropic tests and incubated in thermostat room under continuous light condition (50 µmole photons m^−2^ s^−1^ light intensity, 22 °C). The rate of root bending was determined by measuring the angle formed between the growth direction of root tip and horizontal baseline by ImageJ (NIH, Bethesda, MD, USA, (https://imagej.github.io, accessed on: 10 November 2013) as indicated in the inset, e.g., in Figure 2. Measurements were performed with at least 30 seedlings per each experiment. Three biologically independent experiments were carried out with the same statistical results [35].

### 4.3. Paraquat and Hydrogen Peroxide Tissue Culture Tests

Paraquat (PQ, 1,1-dimethyl-4,4-bipyridinium dichloride or methyl viologen dichloride hydrate, (cat. no.: 856177, Sigma-Aldrich Chemical Co., St. Louis, MO, USA) was used at several concentrations (0–5 µM). The PQ stock solution was prepared by solving PQ in a 1:1 ratio of DMSO and methanol. The H_2_O_2_ (cat.no.: 216763, Sigma-Aldrich Chemical Co., St. Louis, MO, USA) solution (30% *w*/*w* in H_2_O_2_) contained a stabilizer and was used at concentrations of 2–8 mM. Detection of NO in the wild type and mutant Arabidopsis root apex was carried out using seven-day-old plants which were treated with 100 µM sodium nitroprusside (SNP, cat. no.: 71778, Sigma-Aldrich Chemical Co., St. Louis, MO, USA), as a NO donor, or 1 mM 2-4-carboxyphenyl-4,4,5,5-tetramethylimidazoline-1-oxyl-3-oxide (cPTIO, cat. no.: C221, Sigma-Aldrich Chemical Co., St. Louis, MO, USA), as a NO scavenger.

Root gravitropic assays on the different ROS-containing media were essentially performed according to [35] in growth chambers using continuous light conditions (22 °C, 50 µmole photons m^−2^ s ^−1^ light intensity). Upon gravistimulation, the PQ/H_2_O_2_/SNP/cPTIO untreated/treated, vertically grown, 5-to-6-day-old seedlings were reoriented by −135°, and the degree of root curvature was recorded 24 h after rotation by scanning. The rate of root bending was determined by measuring the angle formed between the growth direction of the root tip and the horizontal baseline by ImageJ. The angles were defined according to the inset in Figure 3. At least 70 individual wild type and At*crk5-1* seedlings were tested in a minimum of three separate experiments for PQ and H_2_O_2_ treatments, respectively. For SNP/cPTIO experiments, 6-day-old wild type and At*crk5-1* seedlings (*n* = 30) were grown in MS media as described in4.1. and these seedlings were transferred onto testing media in two separate experiments. Student’s *t*-test was used for statistical analysis for all quantitative measurements.

### 4.4. Histochemical In Situ Detection of ROS

In order to detect the presence of superoxide radical (O_2_^•−^) in the root apex, 6-day-old wild type (AtCol-0) and mutant (At*crk5-1*) seedlings were incubated for 5 min in the staining solution of 300 µM nitroblue tetrazolium chloride (NBT; cat. No.: N5514, Sigma-Aldrich Chemical Co., St. Louis, MO, USA) dissolved in 0.1M Tris-HCl, 0.1 M NaCl, and 0.05 M MgCl_2_, pH = 9.5 based on [53,54]. Quantification of the NBT content in root tips of the wild type and mutant seedlings was based on measuring the pixel intensity of equal squared area (regions of interest, ROI) of the root meristematic zones (MZ) by ImageJ. In situ detection of hydrogen peroxide (H_2_O_2_) was carried out by 3,3′-diaminobenzidine (DAB; cat. no.: D800, SigmaAldrich Chemical Co., St. Louis, MO, USA) in 10-day-old Arabidopsis seedlings according to [79]. Seedlings were imaged under a light stereomicroscope (Nikon SMZ800 (Mitsubishi Corp., Minato, Tokyo, Japan) and they were photographed by a CCD camera (Nikon Coolpix 995, Minato, Tokyo, Japan). Hydrogen peroxide content was quantified using the Invitrogen Amplex^TM^ Red Hydrogen Peroxide/Peroxidase Assay Kit (Thermo Fisher Scientific, cat. no.: A22188, Thermo Fisher Scientific, Waltham, MA, USA) as recommended by the company. Thirty mg fresh plant material was harvested from each sample and ground in liquid nitrogen and diluted in 50 mM potassium phosphate buffer (pH 6.5). The homogenates were centrifuged and the supernatant was used to measure the H_2_O_2_ content. The accumulation of resorufin was determined spectrophotometrically at 560 nm (Thermo Scientific^TM^, Multiscan^TM^ GO Microplate Spectrophotometer, Thermo Fisher Scientific, Vantaa, Finland). The amount of H_2_O_2_ was calculated using a standard curve. Measurements were performed with at least 30 seedlings per each line. Three biologically independent experiments were carried out with the same statistical results.

Nitric oxide levels in Arabidopsis roots were analysed by 4-amino-5-methylamino-2′,7′-difluorofluorescein diacetate (DAF-FM-DA, cat. no.: 251520, Sigma-Aldrich Chemical Co., St. Louis, MO, USA) according to [80]. Seedlings were incubated for 30 min in 10 µM dye solution (prepared in 10 mM Tris–HCl, pH 7.4) and were washed twice within 30 min with Tris–HCl. Samples were analysed using a Zeiss Axiovert 200M-type inverted-fluorescence microscope (Carl Zeiss, Jena, Germany) equipped with a digital camera (Axiocam HR, HQ CCD) and with filter set 10 (excitation 450–490 nm, emission: 515–565 nm). Fluorescence intensities (pixel intensity) were measured on digital images within circular areas of 50 µm radii using Axiovision Rel. 4.8 software (Carl Zeiss, Jena, Germany). The radii of circles were not modified during the experiments. Experiments were carried out three times and 10–12 samples were measured in each experiment.

### 4.5. DR5::GFP and PIN2-GFP Distribution under Oxidative Tests in Roots

In the case of DR5::GFP lines, 6-day-old seedlings from the wild type (DR5::GFP + AtCol-0) and mutant backgrounds (DR5::GFP + At*crk5-1*) were transferred onto PQ lacking (control) and 4 µM PQ half MS media. Then, half of the petri dishes were kept vertically and the other half were immediately rotated by −135°. The petri dishes were kept under continuous light illumination (50 µmole photons m^−2^ s ^−1^ light intensity, 22 °C). The effects of PQ on DR5::GFP signal formation were checked by confocal laser scanning microscopy (CLSM) 4 h–5 h after rotation. For testing the effect of the H_2_O_2_ treatment on DR5::GFP signal distribution, 4 mM hydrogen peroxide was used with the same testing protocol described above for PQ. All experiments were repeated three times.

Testing of the PIN2-GFP signal distribution under oxidative stress generated by PQ was carried out similarly to the above DR5::GFP test, except that the PIN2-GFP signal was monitored by CLSM 5–6 h after rotation. At least 10 seedlings possessing wild type AtCol-0 and At*crk-1* mutant backgrounds for DR5::GFP and PIN2-GFP were tested for root bending tests in the absence/presence of the oxidative stressors, in three independent experiments.

### 4.6. Confocal Laser Scanning Microscopy

For the imaging of auxin distribution in the DR5::GFP and PIN2-GFP constructs in the root gravitropism assays, we used CLSM. The microscopes are located in the ELKH-BRC Cellular Imaging Laboratory, Head: Dr. Gábor Steinbach The DR5::GFP, and the PIN2-GFP expression pattern from the +/- PQ treated and unrotated/rotated seedlings was monitored at different time points upon gravistimulation by an Olympus FV1000 laser scanning microscope system (Olympus, Shinjuku, Tokyo, Japan) with lenses 10×/0.4 Numerical Aperture, and 20×/0.75 Numerical Aperture (Tokyo, Japan). The fluorophore GFP (eGFP) was exited with 488 nm and GFP fluorescence detection was between 505 and 530 nm. The construction of color-coded heat maps to measure fluorescence intensity differences was carried out as described in [35,37,38]. Images were prepared using the Fluo View Ver.3. (Olympus, Shinjuku, Tokyo, Japan)) and Microsoft PowerPoint (Microsoft Corporation, Redmond, WA, USA). Qualification of the fluorescence intensities was carried out by colour-coded heat map construction from Z-stack images (*n* = 10; 10 slices/image) as described in [35,37,38], and the fluorescence intensities were calculated based on pixel intensity measurements by Fiji [81,82]. The average pixel intensity values were measured separately for the left and right sides, and the differences were normalized, calculated from the (L − R)/(L + R) × 2 formula where L = left side and R = right side of the equal square areas superimposed onto the DR5::GFP and PIN2-GFP signals. Values near zero represent a symmetrical signal position, while values higher than 0 mean an asymmetrical signal position. Statistical analyses (two-way ANOVA, means comparisons by Bonferroni) were performed using the OriginPro 2018 software version 9.5 (OriginLab Corporation, Northampton, MA, USA).

### 4.7. Statistical Analysis

The experiments were carried out with two or three independent biological repetitions, as indicated. Sample numbers are given for each experiment in the corresponding Mat&Methods paragraphs. The data are presented as the mean ± standard error (SE) calculated from the combined data of biological repetitions. Statistical analyses (two-way ANOVA, means comparisons by Bonferroni) were performed using the OriginPro 2018 software version 9.5 (OriginLab Corporation, Northampton, MA, USA). Differences of the mutant from the wild type control were determined by Student’s *t*-test and the significant differences were represented as follows: * *p* ≤ 0.1; ** *p* ≤ 0.01; *** *p* ≤ 0.001.

### 4.8. Accession Numbers

Sequence data used in this study can be found in the Arabidopsis Information Resource (TAIR) and GenBank (NCBI) databases under the following accession numbers: AtCRK5 (At3g50530) and AtPIN2 (At5g57090).

## Data Availability

No new data were created or analyzed in this study. Data sharing is not applicable to this article.

## Supplementary Materials

Supplementary materials are available online at https://www.mdpi.com/article/10.3390/ijms22115979/s1.
