# Supplementary material for: The AtCRK5 Protein Kinase Is Required to Maintain the ROS NO Balance Affecting the PIN2-Mediated Root Gravitropic Response in Arabidopsis
Source: Int J Mol Sci. 2021 Jun 1;22(11):5979. doi: 10.3390/ijms22115979 (PMC8197844; doi:10.3390/ijms22115979)
Supplement: Supplementary file 1 [file ijms-22-05979-s001.zip › Supplement ijms-1200716 revised0531.pptx]

## Slide 1
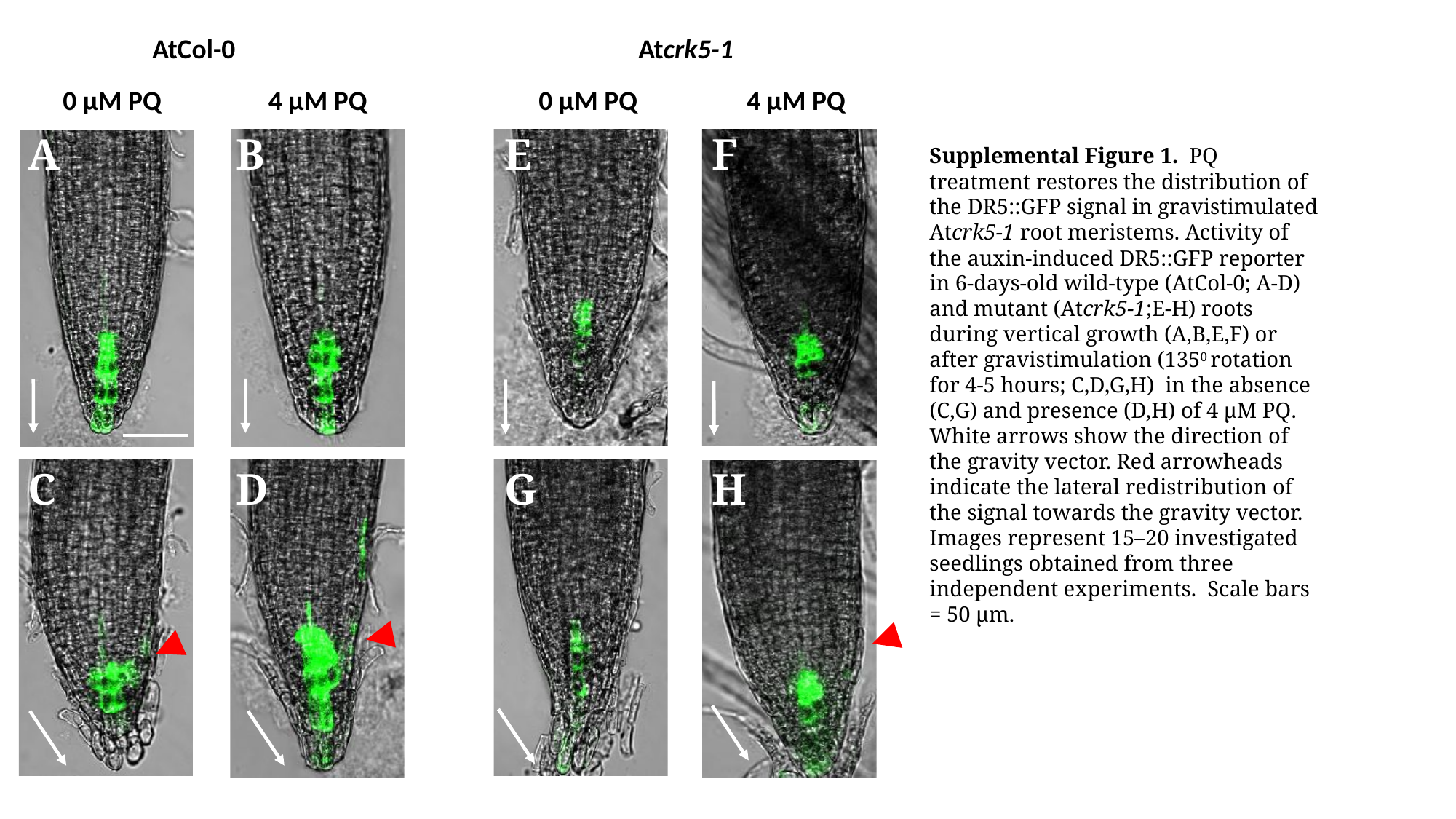

AtCol-0
Atcrk5-1
0 µM PQ
4 µM PQ
0 µM PQ
4 µM PQ
A
B
E
F
Supplemental Figure 1. PQ treatment restores the distribution of the DR5::GFP signal in gravistimulated Atcrk5-1 root meristems. Activity of the auxin-induced DR5::GFP reporter in 6-days-old wild-type (AtCol-0; A-D) and mutant (Atcrk5-1;E-H) roots during vertical growth (A,B,E,F) or after gravistimulation (1350 rotation for 4-5 hours; C,D,G,H) in the absence (C,G) and presence (D,H) of 4 µM PQ. White arrows show the direction of the gravity vector. Red arrowheads indicate the lateral redistribution of the signal towards the gravity vector. Images represent 15–20 investigated seedlings obtained from three independent experiments. Scale bars = 50 µm.
C
D
G
H

## Slide 2
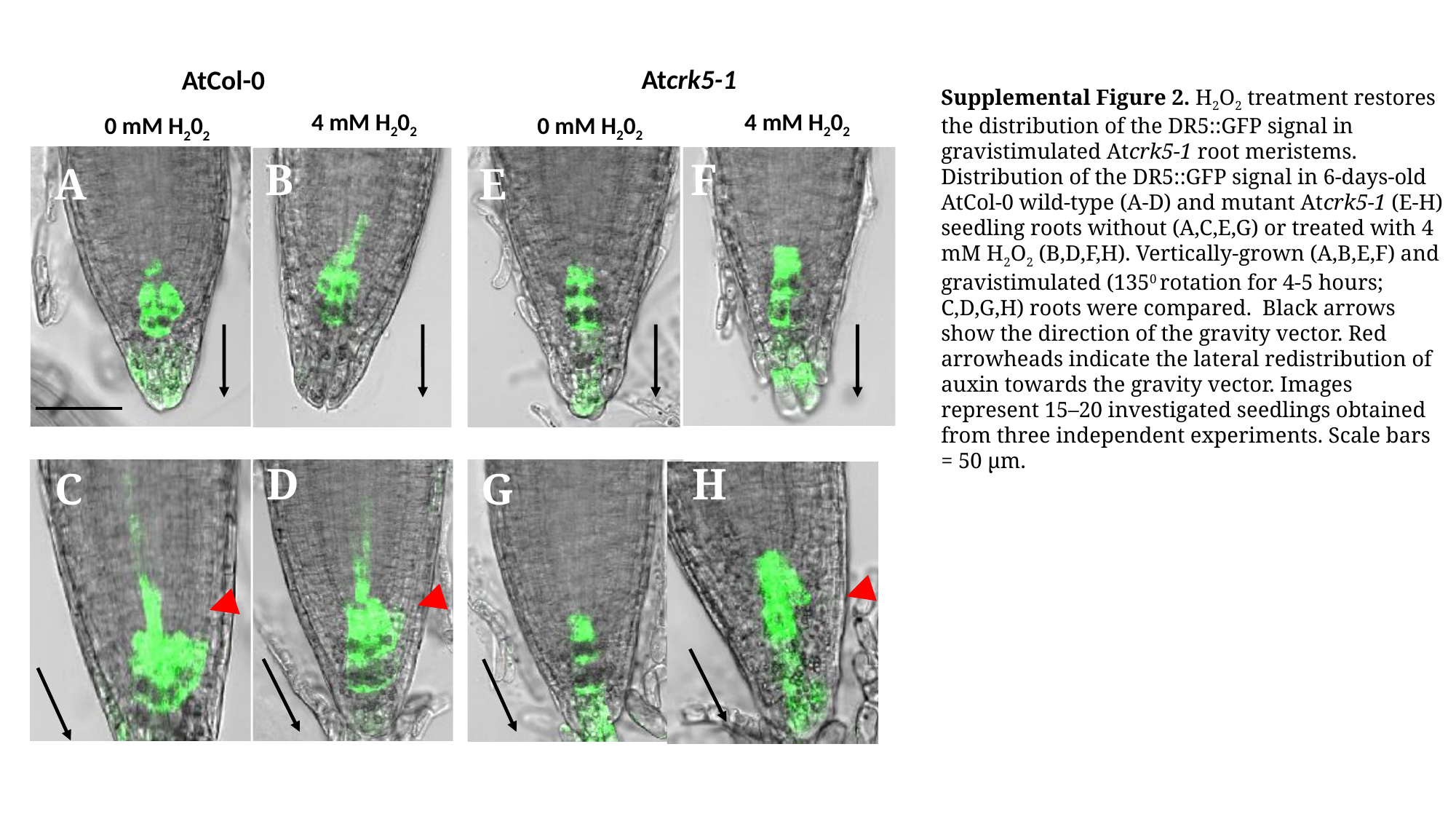

Atcrk5-1
AtCol-0
Supplemental Figure 2. H2O2 treatment restores the distribution of the DR5::GFP signal in gravistimulated Atcrk5-1 root meristems. Distribution of the DR5::GFP signal in 6-days-old AtCol-0 wild-type (A-D) and mutant Atcrk5-1 (E-H) seedling roots without (A,C,E,G) or treated with 4 mM H2O2 (B,D,F,H). Vertically-grown (A,B,E,F) and gravistimulated (1350 rotation for 4-5 hours; C,D,G,H) roots were compared. Black arrows show the direction of the gravity vector. Red arrowheads indicate the lateral redistribution of auxin towards the gravity vector. Images represent 15–20 investigated seedlings obtained from three independent experiments. Scale bars = 50 µm.
4 mM H202
4 mM H202
0 mM H202
0 mM H202
B
F
A
E
D
H
C
G

## Slide 3
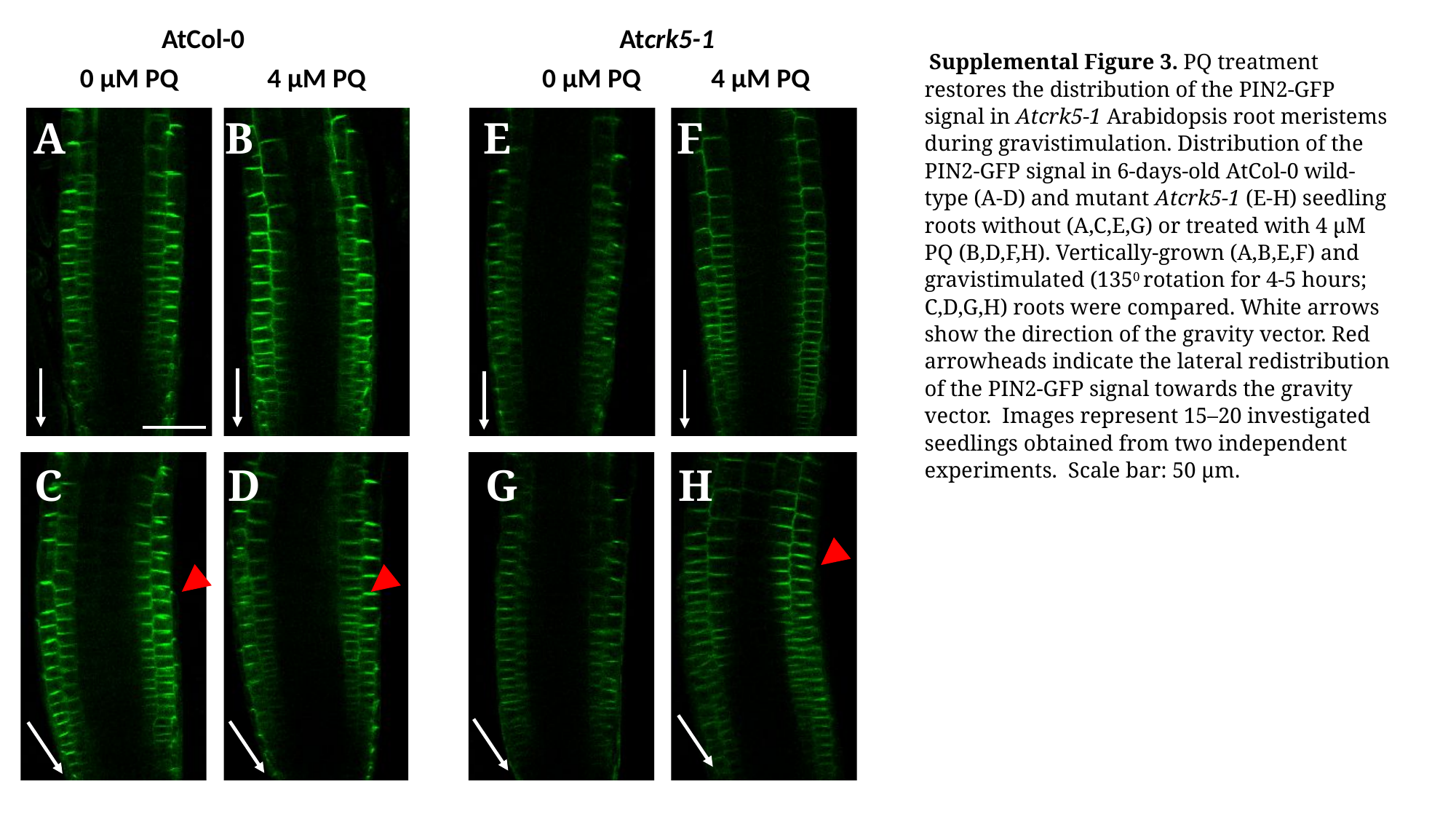

AtCol-0
Atcrk5-1
0 µM PQ
4 µM PQ
0 µM PQ
4 µM PQ
A
B
E
F
C
D
G
H
 Supplemental Figure 3. PQ treatment restores the distribution of the PIN2-GFP signal in Atcrk5-1 Arabidopsis root meristems during gravistimulation. Distribution of the PIN2-GFP signal in 6-days-old AtCol-0 wild-type (A-D) and mutant Atcrk5-1 (E-H) seedling roots without (A,C,E,G) or treated with 4 μM PQ (B,D,F,H). Vertically-grown (A,B,E,F) and gravistimulated (1350 rotation for 4-5 hours; C,D,G,H) roots were compared. White arrows show the direction of the gravity vector. Red arrowheads indicate the lateral redistribution of the PIN2-GFP signal towards the gravity vector. Images represent 15–20 investigated seedlings obtained from two independent experiments. Scale bar: 50 µm.
